# Should We Have Blind Faith in Bioinformatics Software? Illustrations from the SNAP Web-Based Tool

**DOI:** 10.1371/journal.pone.0118925

**Published:** 2015-03-05

**Authors:** Sébastien Robiou-du-Pont, Aihua Li, Shanice Christie, Zahra N. Sohani, David Meyre

**Affiliations:** 1 Department of Clinical Epidemiology and Biostatistics, McMaster University, Hamilton, Ontario, Canada; 2 Population Health Research Institute, McMaster University and Hamilton Health Sciences, Hamilton General Hospital, Hamilton, Ontario, Canada; University of Lille Nord de France, FRANCE

## Abstract

Bioinformatics tools have gained popularity in biology but little is known about their validity. We aimed to assess the early contribution of 415 single nucleotide polymorphisms (SNPs) associated with eight cardio-metabolic traits at the genome-wide significance level in adults in the Family Atherosclerosis Monitoring In earLY Life (FAMILY) birth cohort. We used the popular web-based tool SNAP to assess the availability of the 415 SNPs in the Illumina Cardio-Metabochip genotyped in the FAMILY study participants. We then compared the SNAP output with the Cardio-Metabochip file provided by Illumina using chromosome and chromosomal positions of SNPs from NCBI Human Genome Browser (Genome Reference Consortium Human Build 37). With the HapMap 3 release 2 reference, 201 out of 415 SNPs were reported as missing in the Cardio-Metabochip by the SNAP output. However, the Cardio-Metabochip file revealed that 152 of these 201 SNPs were in fact present in the Cardio-Metabochip array (false negative rate of 36.6%). With the more recent 1000 Genomes Project release, we found a false-negative rate of 17.6% by comparing the outputs of SNAP and the Illumina product file. We did not find any ‘false positive’ SNPs (SNPs specified as available in the Cardio-Metabochip by SNAP, but not by the Cardio-Metabochip Illumina file). The Cohen’s Kappa coefficient, which calculates the percentage of agreement between both methods, indicated that the validity of SNAP was fair to moderate depending on the reference used (the HapMap 3 or 1000 Genomes). In conclusion, we demonstrate that the SNAP outputs for the Cardio-Metabochip are invalid. This study illustrates the importance of systematically assessing the validity of bioinformatics tools in an independent manner. We propose a series of guidelines to improve practices in the fast-moving field of bioinformatics software implementation.

## Introduction

In the last fifteen years an explosion of online accessible bioinformatics tools have occurred in genomics [1]. More recently, the determination of the human genome SNP map through the International HapMap Consortium coupled with the development and commercialization of new methods for high throughput genotyping using SNP microarrays has led to the emergence of genome-wide association studies (GWAS) [2, 3]. According to the HuGE Navigator website (www.hugenavigator.net), 3,253 GWAS have been published up to December 30, 2014 (S1 Fig.). SNAP, a post-GWAS web-based tool, has been developed to find single nucleotide polymorphisms (SNPs) or their proxies and retrieve their annotations in various commercially available genotyping arrays [4]. Additional applications of SNAP include calculating linkage disequilibrium (LD) between SNPs, generating graphical plots of regional associations or LD using data from the HapMap or the 1000 Genomes Project [4]. Since its publication in 2008, SNAP has gained popularity and has been cited 402 times in total with 101 of them being cited in 2014 (according to Web of Science, S2 Fig.). However, despite this growing popularity, little is known about the validity of SNAP outcomes.

Recently, we investigated the early contribution of SNPs to cardio-metabolic traits which have been previously identified in adults through GWAS. Specifically, we were funded to investigate the parental and offspring’s impact of predisposing SNPs on body mass index (BMI), blood pressure (systolic and diastolic blood pressure), fasting glucose, and lipid levels (total cholesterol, low-density lipoprotein cholesterol, high-density lipoprotein cholesterol and triglycerides) traits, in children from the FAMILY birth cohort [5]. We used SNAP to examine whether these SNPs were available on the Illumina Cardio-Metabochip and for those not available on the chip, to find an adequate proxy available on the chip using data from the 1000 Genomes Project. Incidentally during this process, we noticed discordances in the SNAP outputs, which prompted us to formally test the validity of the SNAP software with the Illumina Cardio-Metabochip product file which uses the chromosome and chromosomal position from the NCBI Genome Browser as an independent standard method of SNP selection (inter-method validity).

## Methods

### SNP selection

We selected SNPs that reached genome-wide significance (*P*<5x10^-8^) with body mass index (BMI), blood pressure (systolic and diastolic blood pressure), fasting glucose, and lipid levels (total cholesterol, low-density lipoprotein cholesterol, high-density lipoprotein cholesterol and triglycerides) in populations of European ancestry. We used three different approaches to optimize the SNP selection procedure using a key word search (e.g. BMI) on i) the National Human Genome Research Institute (NHGRI) GWAS Catalog (www.genome.gov/gwastudies/); ii) the HuGE Navigator GWAS Integrator (www.hugenavigator.net/HuGENavigator/gWAHitStartPage.do); iii) the PubMed database (www.ncbi.nlm.nih.gov/pubmed). We identified 69 SNPs associated with BMI, 37 SNPs associated with blood pressure, 39 SNPs associated with fasting glucose level and 276 SNPs associated with lipid levels. Six of the 421 SNPs were pleiotropic which meant they were associated with more than one metabolic trait. These SNPs were only counted once in the overall calculations. A full description of the 415 SNPs, based on the dbSNP version 37 reference, is provided in the S1 Table.

### Assessment of validity

To assess the validity of the SNAP output for the 415 selected SNPs, we checked their availability on the Illumina Cardio-Metabochip (San Diego, CA, USA). This array was designed by seven consortia and consisted of SNPs related to cardiac, metabolic and anthropometric traits. A total of 196,725 SNPs for 23 different traits are available. The design and SNP selection of the array are detailed elsewhere [6].

Two different methods have been used to examine the availability of the subset of 415 SNPs in the Illumina Cardio-Metabochip array. First, we used SNAP and followed the instructions given on their website (www.broadinstitute.org/mpg/snap/). In brief, we used the rs number of each SNP (dbSNP version 37) as the query input and the European sample in HapMap 3 release 2 as the reference [7]. We then applied the filter for Illumina Cardio-Metabochip to look for the availability of the SNP in this specific array. We also used the 1000 Genomes Project European ancestry in addition to the HapMap 3 release 2 reference. SNAP’s outputs for both references on each trait are given in the S2 Table. Both outputs from HapMap 3 and 1000 Genomes Project were individually compared against the Illumina Cardio-Metabochip product file to assess the validity of the SNAP software [8]. Second, we searched the 415 SNPs using their chromosome and chromosomal physical positions from the NCBI Human Genome Browser (Genome Reference Consortium Human Build 37) to ascertain whether SNPs were available in the product file of the Illumina Cardio-Metabochip array (www.illumina.com). The Illumina file provides the chromosome and chromosomal position for each SNP present on the chip. In this file the rs numbers are not available for all SNPs. Using chromosome and chromosomal positions allowed us to avoid discordances between the Illumina file and SNP databases (e.g. incorrect rs number, SNP with several rs numbers).

SNPs that were not available on SNAP, but in fact present in the Illumina Cardio-Metabochip product file were considered as ‘false negative’. Conversely, SNPs specified as available on the Cardio-Metabochip by SNAP, but not by the Cardio-Metabochip Illumina file were classified as ‘false positive’.

### Statistical analysis

The validity between the SNAP output and the Illumina Cardio-Metabochip product file was assessed using Cohen’s Kappa coefficient which calculates the percentage of agreement between both methods [9]. Cohen’s kappa for categorical items is calculated as below:

$$Κ=\frac{\mathrm{Pr}(a)-\mathrm{Pr}(e)}{1-\mathrm{Pr}(e)}$$

Where Pr(*a*) is the observed percentage of agreement and Pr(*e*) is the overall probability of random agreement. We used thresholds established by Landis and colleagues [10], which characterized kappa coefficients over 0.81 as perfect, 0.61–0.80 as substantial, 0.41–0.60 as moderate, 0.21–0.40 as fair, 0–0.20 as slight, and below 0 as having no agreement.

The false negative rate (FNR) was calculated using the number of false negative SNPs dividing by the total number of SNPs tested in each trait. The overall FNR used the 415 SNPs as denominator. The formula is calculated as below:

$$FNR=100\times \frac{FalseNegative}{\left(TruePositive+FalseNegative\right)}$$

The false positive rate formula is calculated as below:

$$FPR=100\times \frac{FalsePositive}{\left(FalsePositive+TrueNegative\right)}$$

## Results

Using the HapMap 3 reference, we found that 9 SNPs out of the 37 SNPs associated with blood pressure traits were not found using SNAP but were available in the Cardio-Metabochip according to the product file (24.3% of false-negatives). This was considered a moderate agreement between both methods as estimated by the Cohen’s Kappa coefficient (Ƙ = 0.469; Table 1, S1 Table). Regarding the fasting glucose-associated SNPs, 19 out of the 39 SNPs were identified by our independent standard method but were not captured by SNAP. The Cohen’s Kappa coefficient indicated no agreement between the two methods (Ƙ = 0; Table 1) and a FNR of 48.7% (Table 1, S1 Table). From a total of 69 BMI-associated SNPs, 34 were available in the Cardio-Metabochip product file but not found by SNAP (49.3% of false-negative). The Cohen’s Kappa coefficient indicated only a slight agreement between the methods (Ƙ = 0.166; Table 1). Regarding the 276 lipid-associated SNPs, 91 out them were not captured by SNAP but were present on the Cardio-Metabochip according to the Illumina product file (FNR = 33%). The Cohen’s Kappa revealed a fair agreement between both methods (Ƙ = 0.295; Table 1). Analyzing all the SNPs together using the HapMap 3 reference in SNAP, we found a Cohen’s Kappa coefficient of 0.250, indicating a fair agreement between the two methods. Overall, 152 out of the 415 SNPs were found by our independent standard method but missed by SNAP representing 36.6% of FNR (Table 1, S1 Table). We did not find any ‘false positive’ SNPs, i.e. SNPs which were present in the SNAP output but missing in the Cardio-Metabochip Illumina’s file when we used the HapMap 3 reference.

**Table 1:** Summary of discordances between outputs from SNAP (the HapMap 3 reference) and the Illumina Cardio-Metabochip file.

Trait	Number of SNPs	Concordances	Discordances	Cohen’s Kappa
Blood pressure	37	28 (75.7%)	9 (24.3%)	0.469
Fasting glucose	39	20 (51.3%)	19 (48.7%)	0.000
BMI	69	35 (50.7%)	34 (49.3%)	0.166
Lipids	276	185 (67.0%)	91 (33.0%)	0.295
TOTAL	415	263 (63.4%)	152 (36.6%)	0.250

Using the 1000 Genomes Project reference in the SNAP workflow, the Cohen’s Kappa coefficient indicated a perfect agreement with the Illumina product file for SNPs associated with blood pressure traits (Ƙ = 0.841; Table 2, S1 Table). Of the 37 SNPs, only 2 were not found by SNAP, corresponding to a FNR of 5.4%. A perfect agreement was also found between the two methods for the SNPs associated with fasting glucose (Ƙ = 1; Table 2). In the subset of 39 glucose-associated SNPs, SNAP gave no false-negative results (Table 2, S1 Table). Regarding the BMI trait, 27 out of 69 SNPs (39.1%) were indicated as missing by the SNAP output while being actually present on the Cardio-Metabochip. A fair agreement between both methods was observed (Ƙ = 0.242; Table 2). We found that 45 out of 276 lipid-associated SNPs were not captured by SNAP but were in fact present on the Cardio-Metabochip as identified by the Illumina product file, representing a false-negative rate of 16.3%. For this subset of SNPs, the Cohen’s Kappa coefficient evidenced a moderate agreement between both methods (Ƙ = 0.525; Table 2). Overall, the use of 1000 Genomes project as reference in SNAP revealed a moderate agreement between the SNAP and chromosomal location methods (Ƙ = 0.487) with 17.6% of false-negatives results. This corresponded to 73 out of 415 SNPs not found on the chip (Table 2, S1 Table). Again, no ‘false positive’ SNP was identified using the 1000 Genomes reference.

**Table 2:** Summary of discordances between outputs from SNAP (the 1000 Genomes reference) and the Illumina Cardio-Metabochip file.

Trait	Number of SNPs	Concordances	Discordances	Cohen’s Kappa
Blood pressure	37	35 (94.6%)	2 (5.4%)	0.841
Fasting glucose	39	39 (100%)	0 (0%)	1.000
BMI	69	42 (60.9%)	27 (39.1%)	0.242
Lipids	276	231 (83.7%)	45 (16.3%)	0.525
TOTAL	415	342 (82.4%)	73 (17.6%)	0.487

## Discussion

In this study, we tested the inter-method validity of SNAP with an independent standard procedure that extracted SNP chromosome and chromosomal positions from the Illumina Cardio-Metabochip product file. Our independent procedure proposes to 1) download the chip product file from the manufacturer’s website; 2) find the physical location of each SNPs (i.e. chromosome and chromosomal position) using an online browser (e.g. dbSNP); 3) check the availability of each SNP in the product file using chromosome and chromosomal positions; 4) if the chromosome and chromosomal positions match with a SNP available in the chip, it means the SNP is available in the chip. We demonstrated that the SNAP outputs for the Cardio-Metabochip were invalid. We used chromosome and chromosomal positions rather than rs numbers to examine whether the SNPs were available on the Cardio-Metabochip array in SNAP. This precludes the possibility that outdated SNP databases used in SNAP (the HapMap 3 release 2 or the 1000 Genomes Pilot 1 versions) are the cause of an improper SNP selection. We propose that the incomplete SNP selection provided by SNAP may result from intrinsic errors in the SNP annotations uploaded for the Cardio-Metabochip array. It is however difficult to confirm this hypothesis as the SNAP source codes are not available for users. In addition, SNAP relies on another software developed by the Broad, Gene Cruiser (www.genecruiser.broadinstitute.org/genecruiser3/), to extract data related to SNP annotations [11]. Looking at the differences of Cohen’s Kappa coefficients using the HapMap 3 and 1000 Genomes references, we speculate that a dysfunction in the SNAP workflow may account for the discordances observed between the two methods. When a list of SNPs is submitted to SNAP, the software first checks whether the SNPs are present in the selected database (HapMap 3 or 1000 Genomes) and then interrogates the availability of the SNPs in the studied array(s). A direct consequence of this counter-intuitive workflow is that a SNP available in the array(s) but absent from the selected database will be considered as missing in the SNAP output giving specific warning messages (*e*.*g*. ‘*WARNING Query snp not in HapMap3_r2*’ or ‘*WARNING Query snp not in 1000GenomesPilot1*’) even if the SNP is available in the chip (S1 Table and S2 Table). The origin of the warning message is dual: 1) query SNP not on any selected array; 2) no LD data available for the SNP in the selected reference database. In both cases however, SNPs are notified a warning message and are considered as missing despite being in fact available on the Illumina Cardio-Metabochip array. It may be more relevant to start the procedures by looking at the SNP availability in the selected array, and then to provide proxies using either the HapMap 3 or the 1000 Genomes references. The validity of SNAP outputs was investigated using diverse metabolic traits in this study. However, it should be noted that we only used the Illumina Cardio-Metabochip and we therefore cannot comment about the general performance of SNAP when other genotyping arrays are queried. We think that this report may encourage the scientific community to double-check SNP selections from SNAP on other genotyping arrays. It must be emphasized that 37 different arrays can be interrogated on the SNAP website (www.broadinstitute.org/mpg/snap/).

Beyond the inaccuracy of the SNAP outputs, another concern is the absence of regular update of the arrays and SNP databases implemented in the software. The last update occurred in October 2012. As novel SNP databases (e.g. latest 2014 release of the 1000 Genomes Project) and DNA chips (e.g. Illumina HumanCore Beadchip or Affymetrix Axiom Biobank arrays) have been released, it is now critical for the SNAP team to offer the best up to date tool to the scientific community. The lack of update of SNAP may not be such a concern if this bioinformatics tool was no longer used by the scientific community. However, since the original article by Johnson *et al*. has been published in 2008, it has reached a peak of citations in 2013 (N = 102; S2 Fig.) [4]. This trend reflects a constantly increasing number of SNAP users since its publication.

We demonstrate that the SNAP outputs for the Cardio-Metabochip are invalid. Based on our results, we strongly encourage SNAP designers [4] to fix the informatics bugs in a timely manner and to inform the scientific community about the updates on their website. In the meantime, we warn readers to avoid using SNAP as the only approach in selecting SNPs from specific arrays as it may produce incorrect outputs and significantly lower the quality of their study design. Researchers can use our alternative method to select SNPs of interest on DNA microarrays (e.g. SNP chromosome and chromosomal physical position on the NCBI Human Genome Browser). We also believe that the growing popularity of the web-based tool SNAP may encourage the SNAP development team to update the software using the more recent genotyping arrays and SNP references.

This study provides an opportunity for reflection on the development of bioinformatics software in an academic setting. Thirty years ago, most of the computing work done by scientists reached high standards of quality. But as the field of computational sciences has become more complex, scientists have hit a ‘steep learning curve’ [12]. The lack of application of high-quality standards in programming can not only cause researchers to waste valuable time and energy but may eventually lead them to produce wrong data and to retract papers, some of them in high-impact journals like *Science* or *Proceedings of the National Academy of Sciences* [13]. The SNAP story is therefore not an isolated event in literature. In this context, actions are warranted to improve the quality of research in bioinformatics, and changes may occur in a variety of ways.

Programmers in private sector have elaborated rigorous methodologies and guidelines (e.g. waterfall model) for developing software, which have evolved considerably during recent years [14]. These gold standard approaches are well-justified in the context of heavily-funded projects involving hundreds of developers and severe financial liabilities or health damage in case of a software failure. However, they have been criticized as too rigid in many other situations including the development of bioinformatics software in public sector, especially when funding is limited and sometimes erratic [14]. Lighter software development models have been proposed, like the Agile, Scrum, XP and Crystal methods, offering interesting alternatives to develop straightforward bioinformatics tools in the academic sector [14]. Despite the wide availability of these resources, high-impact journals in bioinformatics and computational biology do not propose guidelines for software development, in contrast with other disciplines (e.g. epidemiology). Applying guidelines for programming may strengthen the quality of research in bioinformatics as it has already been done in other fields [15].

Improving the computing skills of scientists and bringing trained computer scientists into research groups may avoid researchers to modify existing ‘monster codes’ they do not properly understand, and may help them to simplify and annotate programs to make them more accessible to other users [12, 13].

Unexpectedly, researchers in bioinformatics do not systematically test or document their programs rigorously, and they rarely release their codes, making it almost impossible to reproduce and test [12]. This concern has been identified in the scientific community a long time ago, and as an illustration the *Journal of Money*, *Credit and Banking* requests to upload source codes and data to an archive since 1996 [16]. However, among five leading journals in bioinformatics and computational sciences (*Computational Molecular Biology*, *Bioinformatics*, *Briefings in Bioinformatics*, *PLOS Computational Biology*, *BMC Bioinformatics*), only one journal (*PLOS Computational Biology*) requests that ‘the source code must be accompanied with documentation on building and installing the software from source, as well as for using the software, including instructions on how a user can test the software on supplied test data’ (http://www.ploscompbiol.org/static/guidelines#software). Such requirements are not expected to solve all the problems, but if they are widely adopted by bioinformatics journals they have a great potential to improve transparency in research and enable peers to reproduce and test software [16]. Beta-testing using a double-blind procedure by an independent research team may be included in the original paper and may become a pre-requisite for publication. Initiatives by independent teams to test the validity of bioinformatics software following their publication may be rewarded by the opportunity to publish their conclusions in dedicated ‘beta-test’ sections of bioinformatics journals.

Journals may also compel the designers of bioinformatics tools to perform regular updates under penalty of paper retraction. These updates may be mandatory for a minimum period of time (e.g. 5-years) and may be carried on further depending on the popularity of the software (measured by a website use counter or by citations of the original article by peers).

The Agile Manifesto states that releasing multiple incrementally improved versions of software is more realistic than trying to get everything right the first time [17]. Access to ‘back report’ interfaces on the software’s website by the users may facilitate interactions with designers, helping to collect feedback and include relevant suggestions in subsequent releases [17].

In conclusion, this study demonstrates that the SNAP outputs for the Cardio-Metabochip are often spurious. Besides the discrepancy found in the SNAP web-based tool, this project underscores more general issues observed in bioinformatics field, such as the absence of accessibility of source codes to detect and interpret invalid outcomes or the lack of regular software’s updates. We propose a series of guidelines to improve practices in the fast-moving field of bioinformatics.

## Supporting Information

S1 Fig Number of GWAS publications from 2003 to 2014. (DOCX)

S2 Fig Citations of the paper by Johnson *et al*. (*Bioinformatics*, 2008) since its publication (Web of science, December 30, 2014). (DOCX)

S1 Table SNP by SNP comparison of the SNAP outcomes with a second method based on individual SNP chromosome and chromosomal locations on the Illumina Cardio-Metabochip product file. (XLSX)

S2 Table SNAP’s outputs for both references (HapMap 3 and 1000 Genome Project) and each trait (XLSX)
