# Supplementary material for: Should We Have Blind Faith in Bioinformatics Software? Illustrations from the SNAP Web-Based Tool
Source: PLoS One. 2015 Mar 5;10(3):e0118925. doi: 10.1371/journal.pone.0118925 (PMC4351168; doi:10.1371/journal.pone.0118925)
Supplement: S1 Fig — (DOCX) [file pone.0118925.s001.docx]

**Figure S1. Number of GWAS publications from 2003 to 2014.**

Number of GWAS publications across the years reported by the HuGE Navigator website (December 30, 2014).
