# Supplementary material for: Should We Have Blind Faith in Bioinformatics Software? Illustrations from the SNAP Web-Based Tool
Source: PLoS One. 2015 Mar 5;10(3):e0118925. doi: 10.1371/journal.pone.0118925 (PMC4351168; doi:10.1371/journal.pone.0118925)
Supplement: S2 Fig — (DOCX) [file pone.0118925.s002.docx]

**Figure S2. Citations of the paper by Johnson et al. (Bioinformatics, 2008) since its publication (Web of science, December 30, 2014).**
